# Pro-dopaminergic pharmacological interventions for anhedonia in depression: protocol for a living systematic review of human and non-human studies

**DOI:** 10.12688/wellcomeopenres.19870.1

**Published:** 2023-10-05

**Authors:** Edoardo G. Ostinelli, Virginia Chiocchia, Malcolm Macleod, Michael Browning, Catherine Harmer, Spyridon Siafis, Claire Stansfield, Claire Friedrich, Simonne Wright, Tanatswa Chikaura, Lea Milligan, James Thomas, Carmen Moreno, Toshi A. Furukawa, Soraya Seedat, Jennifer Potts, Georgia Salanti, Andrea Cipriani, GALENOS Team

**Affiliations:** 1 Oxford Precision Psychiatry Lab, NIHR Oxford Health Biomedical Research Centre, Oxford, UK; 2 Department of Psychiatry, University of Oxford, Oxford, England, UK; 3 Oxford Health NHS Foundation Trust, Oxford, England, UK; 4 Institute of Social and Preventive Medicine, University of Bern, Bern, Switzerland; 5 Centre for Clinical Brain Sciences, The University of Edinburgh, Edinburgh, Scotland, UK; 6 Department of Psychiatry and Psychotherapy, Technical University of Munich, Munich, Germany; 7 EPPI Centre, Social Research Institute, University College London, London, England, UK; 8 Department of Psychiatry, Stellenbosch University, Stellenbosch, Western Cape, South Africa; 9 MQ Mental Health Research, London, UK; 10 Department of Child and Adolescent Psychiatry, Institute of Psychiatry and Mental Health, Hospital General Universitario Gregorio Marañón, IiSGM, CIBERSAM, ISCIII, School of Medicine, Universidad Complutense de Madrid, Madrid, Community of Madrid, Spain; 11 Department of Health Promotion and Human Behavior / School of Public Health, Kyoto University, Kyoto, Kyoto Prefecture, Japan

**Keywords:** GALENOS; dopamine; neurotransmitters; anhedonia; depression.

## Abstract

**Background:** Anhedonia is a key symptom of depression, and it has been suggested as a potential target for future individualised treatments. However, much is unknown about how interventions enhancing dopaminergic pathways may affect anhedonia symptoms in the context of depression.

**Methods:** We will perform independent searches in multiple electronic databases to identify clinical and animal experimental studies on pro-dopaminergic interventions in individuals with depression or animal models for depression. The primary outcomes will be overall anhedonia symptoms and their behavioural proxies in animals. Secondary outcomes will include side effects and neurobiological measures. At least two independent reviewers will conduct the study selection, data extraction, and risk of bias assessments using pre-defined tools according to each record’s study design. We will develop ontologies to facilitate study identification and data extraction. We will synthesise data from clinical and animal studies separately. If appropriate, we will use random-effects meta-analyses, or synthesis without meta-analyses. We will investigate study characteristics as potential sources of heterogeneity. We will evaluate the confidence in the evidence for each outcome and source of evidence, considering the summary of the association, potential concerns regarding internal and external validity, and reporting biases. When multiple sources of evidence are available for an outcome, we will draw an overall conclusion in a triangulation meeting involving a multidisciplinary team of experts. We plan updates of the review every 6 months, and any future modifications to the protocol will be documented. We will co-produce this review with multiple stakeholders.

**PROSPERO registration:** CRD42023451821

## Introduction

### Background

Anhedonia is a severe condition characterised by markedly reduced interest or motivation across multiple domains of pleasure [ APA, 2015; Trøstheim *et al.*, 2020]. It is a core feature of depression and a key diagnostic criterion of depressive episodes [ APA, 2015; Elhai *et al.*, 2012]. Self-reported anhedonia is considered a robust predictor of a poorer course of depressive symptoms over time and a lower response to pharmacological treatments [ Morris *et al.*, 2009; Uher *et al.*, 2012]. Although anhedonia is often reported in the context of depression, it is not depression-specific, and it may partially overlap with other mental health conditions [ Trøstheim *et al.*, 2020].

Dopaminergic signalling increasingly gained attention as a promising target candidate to ameliorate anhedonia in people with a diagnosis of depression, although the precise neurobiological mechanisms of anhedonia in major depression are still poorly understood [ Treadway & Zald, 2011]. The use of behavioural paradigms and computational models previously linked to dopaminergic signalling in animal models and human subjects suggested dopamine-related deficits in patients with depression and anhedonia [ Cooper *et al.*, 2018]. Proposed frameworks for dopamine-related deficits in patients with depression and anhedonia are reinforcement learning and effort-based choice evaluating reward processing and motivational deficits [ Cooper *et al.*, 2018].

Overall, anhedonia is a core symptom of depression and qualifies as a promising target for individualised therapies in the future. However, much is unknown about the mechanisms through which dopaminergic pharmacological treatments affect anhedonia symptoms’ severity in the context of depression. Disentangling the potential mechanisms underlying the effect of pharmacological interventions on anhedonia in people with depression is essential to improve current and future care. Moreover, it will lead to a better understanding of the interaction between pharmacological treatments and sub-components of reward processes, providing insight into treatment personalisation. Finally, it can foster the discovery of other interventions to target these mechanisms.

### Review objective(s)

□ To review the evidence on the effect of pro-dopaminergic pharmacological interventions on anhedonia and reward-related tasks in depression.

### Research question(s)

***Animal and pre-clinical studies***

1. Do pro-dopaminergic pharmacological interventions lead to changes in anhedonic behaviours in animal studies, and under what circumstances does this occur?
2. Do the effects of pro-dopaminergic pharmacological interventions on anhedonic behaviour correlate with effects on non-behavioural outcomes?
3. What are the reported side effects of pro-dopaminergic pharmacological interventions in pre-clinical animal experiments of depression?
4. Where a causal pathway (or pathways) may be hypothesised based on the findings of the aforementioned research questions in earlier iterations of this living systematic review, is there any direct evidence to support this hypothesis?

***Human studies***

1. What are the effects of pro-dopaminergic interventions on anhedonia symptom severity in people with depression?
2. What are the effects of pro-dopaminergic interventions on acceptability in people with depression?
3. What are the effects of pro-dopaminergic interventions on tolerability and side-effects in people with depression?
4. What are the effects of pro-dopaminergic interventions on reward- and reinforcement-related tasks in people with depression?
5. What are the effects of pro-dopaminergic interventions on anxiety symptom severity in people with depression?
6. Where a causal pathway (or pathways) may be hypothesized based on the findings of the aforementioned research questions in earlier iterations of this living systematic review, is there any direct evidence available to support this hypothesis?

## Protocol

### Study inclusion and exclusion criteria

***Animal and pre-clinical studies inclusion and exclusion criteria***

Because of the variety of methods used to model aspects of depression in animals and the observation that many of these approaches are used by other researchers without specifically asserting their intention to model depression, we will not require that any specific ‘model of depression’ has been used; but rather seek studies which report some measure of mammalian anhedonic behaviour and which either (a) report the testing of interventions with a recognised pro-dopaminergic mechanism of action (as defined below) or (b) in the absence of a pro-dopaminergic intervention, nonetheless measure dopamine-related non-behavioural outcomes (defined below, including brain imaging; monoamine neurotransmitter concentration; extracellular dopamine concentrations) and anhedonic behaviour in the same animal cohort. Please refer to Table 1 for further details.

**Table 1.** Inclusion and exclusion criteria for animal and preclinical studies.

Study design	We will include: controlled pre-clinical animal experiments investigating pro-dopaminergic pharmacological interventions irrespective of the unit of allocation (e.g., individual animals or cage), parallel or crossover design, study duration and other methodological factors related to study quality and risk of biases (e.g., randomisation, blinding of outcome assessment). We will exclude: in vitro and in silico studies, and uncontrolled experiments, for instance experiments where the animal serves only as its own control.
Population	We will include: only non-human studies involving any mammalian species or zebrafish, any experimental cohort, whether naïve or in which an experimental phenotype has been induced. We will exclude: studies involving other species.
Experimental interventions	We will include: pharmacological interventions with a recognised pro-dopaminergic mechanism of action: OR non- intervention studies where dopamine-related non-behavioural outcomes and anhedonic behaviours are reported from the same cohort of animals.
Control interventions	We will exclude: reports which do not include an appropriate (untreated, or unexposed) control group.
Outcomes	We will include: • Change in anhedonic behaviours following dopaminergic manipulation (primary outcome); • Sucrose or saccharin intake or preference tests; • Hedonic taste reactivity; • Intracranial self-stimulation; • Adverse events including death, autonomic, metabolic, endocrine, neuromuscular, sensorimotor, and behavioural disturbance, which can be measured using batteries or other laboratory measurements. Studies will be included regardless of the outcome reported.

***Human studies inclusion and exclusion criteria***

Please refer to Table 2 for further details.

**Table 2.** Inclusion and exclusion criteria for human studies.

Study design	We will include: randomised controlled trials.
Population	We will include: • Participants with unipolar depression, defined as one of the following: above-threshold symptoms on any standardised measure, or a clinical diagnosis of depression with any operationalised criteria; • Participants of any age; • Studies focusing on both unipolar and bipolar depression will be included as long as the proportion of participants with a bipolar depression is less than 20%. The effect of their inclusion will be tested in a sensitivity analysis (see below). Studies including participants with unspecified depression (e.g., not clear whether it is unipolar or bipolar) will be included, but the effect of their inclusion will be tested in a sensitivity analysis (see below). Studies focusing on people with a comorbidity of mental health illness other than mood disorders (e.g., anxiety) will be included, but the effect of their inclusion will be tested in a sensitivity analysis (see below). We will exclude: • Studies focusing on people with a diagnosis of depression and a serious concomitant medical illness; • Studies focusing on people with a diagnosis of schizophrenia; • Studies focusing on women with post-partum depression as it appears clinically different from major depression [ Cooper & Murray 1998].
Experimental interventions	We will include: any pharmacological treatments administered via any route, alone and at any dose, acting on dopamine pathway/signalling in the central nervous system. We will consider eligible interventions with a direct prevalent or significant dopaminergic agonism or partial agonism mechanism of action at the central nervous system (e.g., dopamine 1-5 receptors; vesicular monoamine transporters, VMAT; dopamine transporters, DAT). For instance, we will include those listed by the Neuroscience based Nomenclature (NbN2R, https://nbn2r.com) with the filter pharma cological==”Dopamine”, and other interventions that might have not been included but fulfil the requirements listed above (see the extended data ( Ostinelli *et al*., 2023) for a non-exhaustive list).
Control interventions	We will include placebo-controlled trials. We will exclude active controls.
Outcomes	We will include: • Anhedonia symptom severity (anhedonia-specific scales, anhedonia-specific sub-scales or individual items focusing on anhedonia from standardised rating scales, both observer-rated and self-rated) (primary outcome); • Acceptability (measured as the proportion of drop-outs due to any reason); • Tolerability (measured as the proportion of drop-outs due to any adverse event); • Safety (measured as the proportion of participants with a specific adverse event). We anticipate high variability in how adverse events are reported. We will use the Medical Dictionary for Regulatory Activities (MedDRA, https://www.meddra.org) to harmonise and organise adverse event-related terminology systematically; • Reward- and reinforcement-related tasks (e.g., probabilistic reward task, effort expenditure for rewards task); • Anxiety symptom severity (measured by observer-rated or self-rated standardised scale); Studies will be included regardless of the outcome reported.

We will extract outcome data reported at 8 weeks post-treatment or manipulation for the above-listed outcomes. If information at 8 weeks is not available, we will consider eligible data ranging between 4 and 12 weeks (with preference to the time point closest to 8 weeks and, if equidistant, the longer outcome).

### Study identification

We plan to regularly search and screen for new potentially eligible studies every 3 months.

***Animal and pre-clinical studies***

We will use a conventional search strategy (Ti/Ab/Keyword/MeSH over PubMed (including pre-prints), Web of Science, Scopus and PsycInfo). We will search for unpublished studies in pre-clinical registries (e.g., animalstudyregistry.org, preclinicaltrials.eu).

***Human studies***

The search strategy will be defined in collaboration with the search team. The ontology team will be informed of the search strategy and will help identify additional search terms where possible and relevant. The resulting search strategy will also inform the scope of the ontology (see a brief ontology protocol in Ostinelli *et al*., 2023).

The electronic searches will include the following literature databases: MEDLINE , Web of Science (SCI, SSCI, ESCI and the related conference and book indexes), EMBASE, SCOPUS, PsycInfo, Cochrane library CENTRAL, Biosis previews and International Pharmaceutical Abstracts. Grey literature searches will include ClinicalTrials.gov and WHO International Clinical Trials Registry Platform (WHO-ICTRP). An example of the search strategy for PubMed is provided in the extended data ( Ostinelli *et al*., 2023).

Past systematic reviews and meta-analyses incidentally identified from the searches will be screened for additional articles not identified via the search strategy. The search strings will combine terms related to pharmacological interventions, major depressive disorder and depressive episode, and filter out indexed animal-only studies and non-trial studies. Terms for pharmacological interventions include an extensive range of chemical synonyms, and trade names. The list of pharmacological interventions originated from the Neuroscience based Nomenclature (NbN2R, https://nbn2r.com) with the filter pharmacological==”Dopamine”. The search terms used for depression were inspired by Dean *et al.* (2021). The searches will combine free and indexed terms. Boolean operators will also be utilised. We plan to update the search every 6 months. The search will not be restricted by time or language. We will collaborate with colleagues and experts in evidence synthesis fluent in any languages the starting team is unfamiliar with and see their help to ensure any records will be assessed appropriately.

### Study selection

The selection process of this review will be reported in line with the Preferred Reporting Items for Systematic Reviews and Meta-Analyses (PRISMA) Statement ( Moher *et al.*, 2009). We plan to record the selection process in sufficient detail to complete a PRISMA flow diagram and a 'Characteristics of excluded studies' table. When any identified and potentially eligible full publication is not available or cannot be accessed, we will contact the original authors to provide further information.

***Animal and pre-clinical studies***

Search results will be de-duplicated using the Automated Systematic Search Deduplicator (ASySD) tool ( Hair *et al.*, 2023) and then uploaded to the Systematic Review Facility (SyRF, app.syrf.org.uk) or a free alternative that can perform a similar function (e.g., Rayyan). Each record will undergo at least two independent screening decisions (‘include’ or ‘exclude’), and in the case of disagreement, the record will automatically be offered to a third independent reviewer.

***Human studies***

Once the searches have been conducted, the duplicates will be removed using EPPI Reviewer. Studies will be selected by two reviewers independently using EPPI Reviewer. The titles and abstracts of all the identified studies will be independently examined by two reviewers and classified as “Retrieve” or “Not retrieve”. Full texts of all studies labelled as “Retrieve” will be accessed and screened independently for full eligibility by two independent reviewers. Uncertainty regarding study inclusion will be resolved through discussion with a third review team member.

***Data extraction***

Data will be extracted by two reviewers independently using SyRF (animal and pre-clinical studies) or EPPI Reviewer (human studies). Data extraction will follow an a priori-developed data extraction template. The data extraction form will be sent to the ontology team so that relevant ontology categorisations can be identified to support data extraction. We will collect data on study (e.g., date of completion, date of publication) and population characteristics (e.g., age, sex assigned at birth), intervention, comparator, and outcomes.

For continuous outcomes, we will extract means and standard deviations of pre-intervention and endpoint data (if not available, change from baseline score). Missing standard deviations will be calculated from standard errors and, if not available, it will be obtained as follows: 1) from test statistics; 2) from confidence intervals (CIs); 3) from median/ranges and other measures of distribution; 4) by contacting the original study authors; 5) using a validated imputation method ( Furukawa *et al.*, 2006). Whenever a value is reported but it is unclear whether it represents the standard deviation or the standard error, we will: 1) check this value against other available approaches (see above); 2) contact the original study authors; 3) consider it as a standard error to keep a conservative approach (preferring potential overestimation over underestimation of the true missing standard deviations).

For dichotomous outcomes, we will extract the absolute number of events and the number of total participants allocated to the relevant group; if the latter is not available, we will extract the observed sample. In case a study reports the same outcome using more than one measure (e.g., as continuous, and as dichotomous such as symptoms score and number of responders), we will prioritise the continuous measurement.

Any disagreement will be solved via discussion between the two reviewers and a third reviewer.

Where relevant outcome data are not reported, we will contact the original authors to obtain them. We will report the risk of bias assessment for each study in the extended data of the future publication. The risk of bias assessment will inform the confidence in the evidence (“Summary of the evidence” section).

***Animal and pre-clinical studies***

We will extract all available information for each outcome (e.g., reported correlation/covariance). Where sample size is not adequately reported, this will be estimated where possible (e.g., using the lower boundary of a reported range). We will include outcome data for each behavioural test of anhedonia for each time point tested. Where the intervention consists of only a few doses, and/or the outcome is measured over a short period and a monophasic response is expected (rising to a peak and falling towards baseline) we will calculate the effect size as the difference in areas under the response curves; where this is not the case, we will use the last time point presented.

We will evaluate the completeness of reporting using a modified version of the CAMARADES checklist ( Macleod *et al.*, 2004). The completeness of reporting of study design, conduct and analysis is a pre-requisite for evaluation of risks of bias. Reporting is often incomplete, meaning many publications are graded as having an ‘unclear’ risk of bias across multiple dimensions. We will therefore evaluate the completeness of reporting using an extended version of the ARRIVE10 tool (( Ostinelli *et al*., 2023); including 2 categories from the ARRIVE 2.0 ’Recommended’ rather than ‘Essential’ set). We will evaluate the risk of bias using the SYRCLE tool ( Hooijmans *et al.*, 2014).

For animal studies, the reconciliation process will be managed automatically using an R-shiny tool linked to the SyRF platform. A lower agreement rate is expected with quantitative data extracted from figures; these will be flagged for reconciliation where there is a difference of more than 10% in the values extracted; otherwise, the mean value will be carried forward to analysis.

***Human studies***

For human studies, randomised controlled trials will be assessed with the Risk of Bias 2 (RoB 2.0) tool from Cochrane ( Sterne *et al.*, 2019). RoB 2.0 assesses five domains (risk of bias arising: from the randomisation process, due to deviations from the intended interventions, due to missing outcome data, in measurement of the outcome, and in selection of the reported results) ( Sterne *et al.*, 2019). The overall risk of bias of studies will be graded as a) high risk of bias if they have at least one domain judged as high risk, b) low risk of bias if at most one of the domains was judged as moderate risk and no domains were judged as high risk, c) some concerns about bias for all other cases.

### Data analysis and synthesis

***Comparison of study findings and synthesis***

Statistical analyses will be performed using the *meta* and *metafor* packages in R.

*Animal and pre-clinical studies*

The effect size for continuous outcomes will be the standardised mean difference (SMD; calculated as Hedges g). Because group size in animal studies can be small, and measured variances can be low (or zero), we will conduct sensitivity analyses using normalised mean difference (NMD) effect sizes. Effect size estimates will be corrected for the direction of effect (ie does a higher number on a given scale reflect more or less anhedonic behaviour) by multiplication, where appropriate, by -1 such that a negative effect size represents an improvement in the observed behaviour.

For data synthesis we will use a multivariate multilevel model with meta regression and robust variance estimation ( Yang *et al.*, 2023). We will include random effects covariates for the publication (because this may include several experiments), experiment (because this may include several outcome measures), species, method of induction and outcome measured. Where there are multiple effect sizes for the same animal cohort, we will estimate the within study variance-covariance matrix (VCV) using reported correlations/covariance or, if this is not available, assuming a correlation of ρ = 0.5 (with sensitivity analyses with estimates of 0.2 and 0.8). Other potential sources of heterogeneity will be included as fixed effects covariates.

We will use the restricted maximum likelihood (REML) to estimate between study variance τ and the between study VCV in multivariate meta-analysis models. We will use Hartung-Knapp correction for the 95% CIs if there are at least 5 studies. We will report the τ estimate, its 95% CIs and the prediction interval of the summary effect. If there are insufficient data for quantitative analysis (fewer than 5 included publications or fewer than 10 effect sizes) we will present a qualitative summary of the available evidence and report a synthesis without meta-analysis (SWiM) ( Campbell *et al.*, 2020).

*Human studies*

For outcomes measured on different scales, we will check if their effect is measured in the same direction (a high value for a scale would translate into a lower value at another scale for the same outcome) and translate it where needed to harmonise the direction (e.g., equipercentile linking, score inversion and score centering). For instance, a scale measuring “daily pleasure intensity” will be harmonised to have the same direction of the pre-specified outcome “anhedonia symptom severity”. We plan to use endpoint scores and, if not available, change from baseline scores in a single model if enough data allow such calculation.

If enough comparative data is available for the same outcome, we plan to perform quantitative synthesis via a pairwise meta-analysis of active treatments (grouped) versus placebo and each active treatment versus placebo. We will employ a random-effects model within a frequentist setting. We will assess heterogeneity by visual inspection of forest plots, considering the direction and magnitude of effects and the degree of overlap between CIs. When estimating the heterogeneity variance τ, we will use REML and correct the 95% CIs using a Hartung-Knapp correction if there are at least 5 studies. We will report the τ estimate, its 95% CIs and the prediction interval of the summary effect. For continuous outcomes, we will report the effect as mean difference if the same scale has been used in the studies contributing to the analysis for the outcome of interest or as SMD (calculated as Hedges’ g) if two or more scales provided data of interest. For dichotomous outcomes, we will measure and report the effect as odds ratio (OR). Additionally, we will use the event rate in the placebo group (control event rate, CER), convert the summary meta-analytic OR to relative risk (RR) and calculate the experimental event rate (EER) to facilitate communication of benefit-harm information. If a meta-analysis is not possible, we will follow available guidelines to perform and report a synthesis without meta-analysis (SWiM) ( Campbell *et al.*, 2020).

***Exploration of heterogeneity***

We will examine potential study or population characteristics as source of heterogeneity of treatment effects for the primary outcomes. If a meta-analysis is possible and there are sufficient data, we will perform sub-group analyses (e.g., meta-regression). We will investigate the following variables for both animal and human studies (unless otherwise specified):

- Sociodemographic variables
  - Age
  - Sex assigned at birth
  - Socioeconomic status (human studies only)

- Baseline clinical variables
  - Anhedonia baseline severity
  - Reward baseline sensitivity
  - Anxiety baseline severity
  - Depression baseline severity
  - Quality of life baseline severity (human studies only)

- Pharmacological treatment
  - Dose
  - Duration of treatment

***Sensitivity analyses***

*Animal and pre-clinical studies*

If a meta-analysis is possible, we will explore the robustness of the findings for the primary outcomes by 1) restricting the analysis to studies with an overall low risk of bias; 2) excluding estimates with imputed values.

*Human studies*

If a meta-analysis is possible, we will explore the robustness of the findings for the primary outcomes by 1) restricting the analysis to studies with an overall low risk of bias; 2) excluding from the analysis studies focusing on both unipolar and bipolar depression; 3) excluding from the analysis studies reporting data on unspecified depression; 4) excluding from the analysis studies focusing on mental health comorbidities other than mood disorders (e.g., anxiety).

### Reporting bias

We will examine the selective non-reporting (or under-reporting) of results by evaluating whether outcome data availability is associated with the magnitude or direction of the result. We will also examine small-study effects for primary outcomes with data from at least 10 studies, by visually inspecting contour-enhanced forest plots and via Egger’s regression test. We will assess the potential impact of reporting bias on the magnitude and direction of the findings using the ROB-ME tool ( Page *et al.*, 2021).

## Summary of the evidence

We will assess the certainty of evidence for the primary outcomes and each source of evidence (animal/pre-clinical and human) using an adapted version of the GRADE framework ( Hooijmans *et al.*, 2018; Schünemann *et al.*, 2013). A single reviewer will judge the importance of the biases and the confidence in the evidence, and the judgements will be verified by a second reviewer. Any disagreements will be discussed and a third review team member consulted if necessary. The confidence in the evidence will be assessed by assigning “no concerns”, “some concerns” or “major concerns”. We will present the assessments in summary of evidence (SoE) tables for each primary outcome, by presenting in the rows the different sources of evidence (e.g., animal and human studies) and in the columns the different domains relevant to the confidence of the evidence ( Table 3).

**Table 3.** Summary of Evidence (SoE) table.

Source of evidence	Summary of the association	Internal validity	External validity	Reporting bias and other sources of meta-bias
Animal and pre-clinical studies on change in anhedonic behaviours following dopaminergic manipulation (separately for different comparisons)	Number of studies and total sample size. If a meta-analysis is feasible, numerical summary from the meta-analysis (point estimate, 95% confidence intervals and 95% prediction intervals). If a meta-analysis is not feasible, we will report the numerical SWiM range. Distribution of the effect sizes across the individual studies and their direction.	Percentage of studies with low, moderate, or high risk of bias (see “Risk of bias assessment”). We will consider the overall judgement, the judgements across domains and the potential direction of bias (e.g., towards the null or to any direction). Assessment of the robustness of the findings with a sensitivity analysis restricting to studies with an overall low risk of bias (see “Sensitivity analysis”).	Assessment of the degree to which the characteristics of the included studies reflect the clinical setting. We will also consider the potential direction of the bias in case of indirectness.	Assessment of the potential impact of reporting bias on the magnitude and direction of the findings using the ROB-ME tool ( Page *et al.*, 2021). No other sources of meta-bias are expected, as we will follow a rigorous review methodology aimed at minimizing biases in the review process.
Human studies for the effects on anhedonia symptom severity (separately for different comparisons)				

## Triangulation of the evidence from living systematic reviews

Human and animal studies will produce different types of evidence. However, given that their respective systematic errors and biases are largely unrelated, we will jointly consider their results in terms of about (1) the direction and (2) the strength of evidence to reach an overall conclusion using triangulation methods.

In every update we will assess the potential for triangulation based on the amount of data and the presence of bias. A triangulation meeting will take place when sufficient evidence for at least one outcome is available from at least two sources. We will invite neuropsychopharmacologists and methodologists alongside the review team to the triangulation meeting. The available evidence from all the SoE tables for the same outcome will be described and considered together to draw an overall conclusion about the impact of the intervention/risk factor/exposure on the studied outcome.

## Updating the systematic review and stopping the living mode of the review

### Updating process of the living systematic review

When new eligible studies are included, we plan to incorporate new evidence and update the living systematic reviews based on their potential impact on substantially changing the overall findings. The authors of the last iteration of the living systematic review will make this assessment. We will clearly report any update, highlighting what has been added, and specify when each update has been performed in our product versioning system. We will use a versioning system based on the one used by F1000, and any deviations from the methods outlined in this protocol will be documented and justified. After the conclusion of the first iteration of the systematic review, the methods will be reconsidered to judge their suitability and efficiency in practice. If deemed appropriate and in response to new evidence, the inclusion criteria (e.g., non-placebo comparators) and the methodological approaches of data extraction and synthesis (e.g., network meta-analysis) will be adapted accordingly, and any changes will be documented. For instance, it is likely that future iterations will include genetic as well as pharmacological manipulations of dopaminergic systems.

### End of support to the living mode of the review

The support to the living mode of the review will be assessed at every triangulation meeting. In the event no triangulation meeting happens, the review team will continue supporting the living mode of the review. The living mode will also continue where the triangulation team decides that new, relevant evidence is required to fulfil the review aims. When it is decided that there is a saturation of evidence and that the research aims of the living systematic review have been satisfied, the living mode of the systematic review will be stopped.

## Co-production aspects

We have employed a multidisciplinary approach by considering the perspectives, experience and knowledge of multiple stakeholders such as preclinical and clinical researchers, clinicians, systematic review methodologists, statisticians, and experiential advisors.

In formulating the focus of the review, we utilized existing prioritization exercises that had co-production imbedded in their process, namely, the United Kingdom Mental Health research goals 2020–2023, the WHO Grand Challenges in Mental Health ( Collins *et al.*, 2011) and the James Lind Alliance’s Top 10 Priorities for Depression (2016) and Schizophrenia (2011). Common themes emerged, including research to develop new and improved treatments, root causes, better understanding of therapeutic mechanisms for current drug and psychological treatments, and this is the starting point for the initial living systematic review questions within GALENOS.

To ensure the comprehensive consideration of perspectives from all stakeholders involved, we will assemble a team of co-authors who represent the diverse backgrounds mentioned above. It is anticipated that each co-author will make a more substantial contribution to specific sections based on their individual experiences and expertise. The review team will be provided with guidance by work package 1 on effective models of involvement for Experiential Advisors. As a result, a multidisciplinary approach will be implemented throughout all stages of the review, from the identification of needs, the formulation of the research aims, the design of the review, and the interpretation and dissemination of the findings to the research and public community.

Considering the complexity and multidimensionality of the review topic, we will establish a schedule of regular team meetings and foster effective communication within the GALENOS project. The primary objective of these initiatives is to facilitate a shared understanding, promote the transferability of knowledge, encourage the exchange of ideas and perspectives, and identify the distinct needs of various stakeholders. By implementing these measures, we aim to create an environment where all stakeholders have equal standing and can actively contribute to the collaborative production of the review.

## Dissemination of information

We plan to publish the review on the GALENOS website and on Wellcome Open Research. A Plain English summary will be included in each iteration of the living systematic review. We will disseminate our findings using social media (e.g., Twitter) and the GALENOS blog. We will also include the results in the quarterly Research Roundup newsletter from MQ.

## Study status

The study status at the date of submission [4 ^th^ August 2023] is reported below.

### Preliminary searches

Started, not completed.

### Piloting the study selection process

Not started.

### Piloting the study selection process

Not started.

### Full searches

Not started.

### Full screening of search results against eligibility criteria

Not started.

### Data extraction

Not started.

### Risk of bias or quality assessment

Not started.

### Data synthesis

Not started.

## Ethics and consent

Not applicable.

## Data Availability

No underlying data is associated with this protocol. Open Science Framework: LSR1. https://doi.org/10.17605/OSF.IO/CNB5R ( Ostinelli *et al*., 2023) Data are available under the terms of the Creative Commons Attribution 4.0 International license (CC-BY 4.0). PRISMA-P checklist for ‘Pro-dopaminergic pharmacological interventions for anhedonia in depression: protocol for a living systematic review of human and non-human studies’. https://doi.org/10.17605/OSF.IO/CNB5R ( Ostinelli *et al*., 2023) Data are available under the terms of the Creative Commons Attribution 4.0 International license (CC-BY 4.0).
