# Supplementary material for: Pro-dopaminergic pharmacological interventions for anhedonia in depression: protocol for a living systematic review of human and non-human studies
Source: Wellcome Open Res. 2023 Oct 5;8:425. [Version 1] doi: 10.12688/wellcomeopenres.19870.1 (PMC11255544; doi:10.12688/wellcomeopenres.19870.1)
Supplement: Peer review history

# Reviewer report: Reviewer response for version 1

**DOI:** 10.21956/wellcomeopenres.22004.r80246

**Published:** 2024-07-17

**Authors:** Eric Nunes

**Affiliations:** 1 Yale School of Medicine, New Haven, CT, USA

An approach like this is vital, given the urgent therapeutic need for treatments targeting anhedonia and the intricacies of the construct. The proposed protocol achieves this effectively and is well-justified. It follows the latest standards for systematic reviews and features a compelling multidisciplinary, cross-species approach. The resulting review will likely have a substantial impact on treatments for anhedonia across various health conditions.

 The comments provided below, I believe, will help with this ambitious endeavor.

 Anhedonia, is a multifaceted construct encompassing cognitive, emotional, and physical domains. It's typically divided into two primary components:

- The inability to experience pleasure (often termed "consummatory anhedonia")
- Impaired goal-directed behavior (referred to as "motivational anhedonia" or "avolition").

Dissociating these components is essential for understanding the underlying mechanisms and tailoring interventions for major depressive disorder (MDD).

Consummatory Anhedonia (Inability to Experience Pleasure):

- Refers to the diminished ability to enjoy pleasant stimuli and experiences.
- Assessed through self-report measures (e.g., Snaith-Hamilton Pleasure Scale) that directly inquire about enjoyment and pleasure in response to rewarding stimuli.

Motivational Anhedonia (Impaired Goal-Directed Behavior):

- Reflects a reduction in the drive to engage in activities that would typically be rewarding.
- Assessed through tasks that measure response initiation and effort in anticipation of rewards (e.g., Monetary Incentive Delay Task).

Dissociating the Two Components:

- Behavioral and Experimental Tasks: Different tasks can measure distinct aspects of reward processing. For instance, assessing response to anticipated rewards may help to separate motivational aspects from the actual enjoyment of rewards.
- Neuroimaging: Brain imaging techniques, like fMRI, can reveal differences in brain regions associated with reward anticipation (motivational) versus reward receipt (consummatory).
- Longitudinal and Cross-Sectional Studies: Differentiating between chronic patterns of pleasure loss versus reduced motivation across time can elucidate distinct pathways of anhedonia in individuals.

A clear definition of anhedonia is crucial for shaping study protocols and determining the approach to examining preclinical and clinical literature. 

For example this can guide what outcomes to include:

- Change in anhedonic behaviours following dopaminergic manipulation (primary outcome)
- Sucrose or saccharin intake or preference tests
- Hedonic taste reactivity
- Intracranial self-stimulation

In preclinical research, the use of sucrose or saccharin preference tests as measures of anhedonia is frequently debated due to their limited scope and interpretative clarity. These tests mainly focus on consummatory anhedonia, which is the animal's ability to experience pleasure from sweet substances. However, they do not effectively capture motivational anhedonia, which relates to the drive and motivation to pursue rewarding activities. Including behavioral models that measure motivated behavior and effort-based choice is crucial for a more nuanced understanding of anhedonia and could offer better insights into the effects of dopamine manipulation. See work from Salamone JD, *et al.*, 2024 ^1^.

Table 2. Inclusion and exclusion criteria for human studies.

 Anhedonia extends beyond unipolar depression and affects multiple psychiatric disorders, including bipolar disorder and schizophrenia, where it often correlates with negative symptoms like avolition. Incorporating these populations in studies can provide valuable insights into the different features of anhedonia, such as consummatory versus motivational, and their sensitivity to dopamine manipulation. See work from Treadway MT, *et al.*, 2011 ^2^.

Is the study design appropriate for the research question?

Yes

Is the rationale for, and objectives of, the study clearly described?

Partly

Are sufficient details of the methods provided to allow replication by others?

Yes

Are the datasets clearly presented in a useable and accessible format?

Not applicable

Reviewer Expertise:

With extensive experience in preclinical models of depression, I have focused on understanding the behavioral and neurobiological mechanisms underlying depression-like behavior and motivational deficits. My expertise includes designing and conducting experiments to assess anhedonia, reward processing, and goal-directed behavior.

I confirm that I have read this submission and believe that I have an appropriate level of expertise to confirm that it is of an acceptable scientific standard, however I have significant reservations, as outlined above.

## References

[1] : The Neurobiology of Activational Aspects of Motivation: Exertion of Effort, Effort-Based Decision Making, and the Role of Dopamine. *Annu Rev Psychol* .2024;75: 10.1146/annurev-psych-020223-012208 1-32 10.1146/annurev-psych-020223-012208 37788571

[2] : Reconsidering anhedonia in depression: lessons from translational neuroscience. *Neurosci Biobehav Rev* .2011;35(3) : 10.1016/j.neubiorev.2010.06.006 537-55 10.1016/j.neubiorev.2010.06.006 20603146 PMC3005986

# Reviewer report: Reviewer response for version 1

**DOI:** 10.21956/wellcomeopenres.22004.r81000

**Published:** 2024-05-31

**Authors:** Balwinder Singh

**Affiliations:** 1 Mayo Clinic, Rochester, MN, USA

In this systematic review protocol, the authors aim to identify clinical and animal experimental studies related to pro-dopaminergic interventions in individuals with depression or animal models for depression. The methodology is comprehensive, covering both human and animal studies. However, for one of the aims, investigating the efficacy of interventions at 8 weeks, there’s uncertainty regarding whether ketamine, which has been hypothesized to lead to a hyperglutamatergic state but also exhibits pro-dopaminergic action, should be considered as a pro-dopaminergic intervention. It might be worthwhile to expand the efficacy duration of ketamine to 24 hours to 7 days.

 However, one realistic concern is the limited availability of suitable interventions. Additionally, many studies reporting anhedonia data may have small sample sizes, potentially impacting the overall impact of the systematic review. Nevertheless, it remains a valuable academic exercise

Is the study design appropriate for the research question?

Yes

Is the rationale for, and objectives of, the study clearly described?

Yes

Are sufficient details of the methods provided to allow replication by others?

Yes

Are the datasets clearly presented in a useable and accessible format?

Not applicable

Reviewer Expertise:

Treatment-resistant depression; novel interventions, especially ketamine; biomarkers devlopement; bipolar disorder

I confirm that I have read this submission and believe that I have an appropriate level of expertise to confirm that it is of an acceptable scientific standard, however I have significant reservations, as outlined above.

# Reviewer report: Reviewer response for version 1

**DOI:** 10.21956/wellcomeopenres.22004.r73020

**Published:** 2024-03-08

**Authors:** Erika E Forbes

**Affiliations:** 1 University of Pittsburgh, Pittsburgh, PA, USA

The proposed protocol is timely, clearly written, thorough, and well justified. The approach to the living systematic review adheres to current standards for systematic reviews, and the multidisciplinary approach to the topic and cross-species focus are especially compelling. The resulting review will likely have a meaningful influence on mental health science.

 As there are several examples of non-parallel approaches (or different levels of detail) for animal and human studies, considerations are presented separately below. In general, it would be worthwhile to provide consistent information.

 The following could be addressed in the material on animal and pre-clinical studies:

 1. In the questions guiding review of animal studies, what is meant by "non-behavioral outcomes"? A definition and some examples might be helpful.

 2. Why are zebrafish the only non-mammalian species included?

 3. If studies without control groups will be excluded, are included non-intervention studies defined as other types of experimental studies with a randomized, controlled design?

 4. If sucrose preference and other constructs hypothesized to capture anhedonia are secondary, what is meant by "anhedonic behaviours" in the list of outcomes?

 The following could be addressed in the material on human studies:

 1. For post-treatment time point, what is the rationale for focusing on 8 weeks? Why is 12 weeks the maximum?

 2. Why is anxiety severity included in outcomes?

 3. Why is presence/absence of anhedonia as a symptom (e.g., from structured diagnostic interviews) not included in outcomes?

 A few other aspects of the proposed methods could be clarified:

 1. What are the proposed search terms?

 2. Why will selection of human studies include two steps (retrieve and include) but study selection of animal and pre-clinical studies include only one? The "retrieve" step seems to involve titles and abstracts only, but the relevance to human studies only is unclear.

 3. Will risk of bias in animal studies be graded as with risk of bias in human studies? What are the factors or domains in the SYRCLE tool? How many people will rate risk of bias for each study?  

 The authors are careful to describe the living mode of the review, but the plan is somewhat underdeveloped. Is there an estimated timeline for updating? What are the criteria for determining "that there is a saturation of evidence and that the research aims of the living systematic review have been satisfied"?

 Finally, for those not as familiar (e.g., readers outside the UK), it would be worthwhile to briefly define GALENOS.

Is the study design appropriate for the research question?

Yes

Is the rationale for, and objectives of, the study clearly described?

Yes

Are sufficient details of the methods provided to allow replication by others?

Yes

Are the datasets clearly presented in a useable and accessible format?

Not applicable

Reviewer Expertise:

I am a clinical psychologist, developmental psychopathologist, and affective neuroscientist with expertise in the role of reward neural systems in depression, anhedonia, and related difficulties. My research includes methods such as functional magnetic resonance imaging, ecological momentary assessment, phone sensor recording, and transcranial magnetic stimulation.

I confirm that I have read this submission and believe that I have an appropriate level of expertise to confirm that it is of an acceptable scientific standard.
